# Clinical landscape of LAG-3-targeted therapy

**DOI:** 10.1016/j.iotech.2022.100079

**Published:** 2022-03-17

**Authors:** L. Chocarro, E. Blanco, H. Arasanz, L. Fernández-Rubio, A. Bocanegra, M. Echaide, M. Garnica, P. Ramos, G. Fernández-Hinojal, R. Vera, G. Kochan, D. Escors

**Affiliations:** 1 Oncoimmunology Research Unit, Navarrabiomed-Fundación Miguel Servet, Universidad Pública de Navarra (UPNA), Hospital Universitario de Navarra (HUN), Instituto de Investigación Sanitaria de Navarra (IdiSNA), Pamplona, Spain; 2 Division of Gene Therapy and Regulation of Gene Expression, Cima Universidad de Navarra, Instituto de Investigación Sanitaria de Navarra (IdISNA), Pamplona, Spain; 3 Medical Oncology Unit, Hospital Universitario de Navarra (HUN), Instituto de Investigación Sanitaria de Navarra (IdiSNA), Pamplona, Spain; 4 Medical Oncology Department, Hospital Clínico San Carlos, Madrid, Spain

**Keywords:** LAG-3, immune checkpoint, immunotherapy, targeted therapy, cancer treatment

## Abstract

Lymphocyte-activated gene 3 (LAG-3) is a cell surface inhibitory receptor and a key regulator of immune homeostasis with multiple biological activities related to T-cell functions. LAG-3 is considered a next-generation immune checkpoint of clinical importance, right next to programmed cell death protein 1 (PD-1) and cytotoxic T-cell lymphocyte antigen-4 (CTLA-4). Indeed, it is the third inhibitory receptor to be exploited in human anticancer immunotherapies. Several LAG-3-antagonistic immunotherapies are being evaluated at various stages of preclinical and clinical development. In addition, combination therapies blocking LAG-3 together with other immune checkpoints are also being evaluated at preclinical and clinical levels. Indeed, the co-blockade of LAG-3 with PD-1 is demonstrating encouraging results. A new generation of bispecific PD-1/LAG-3-blocking agents have also shown strong capacities to specifically target PD-1+ LAG-3+ highly dysfunctional T cells and enhance their proliferation and effector activities. Here we identify and classify preclinical and clinical trials conducted involving LAG-3 as a target through an extensive bibliographic research. The current understanding of LAG-3 clinical applications is summarized, and most of the publically available data up to date regarding LAG-3-targeted therapy preclinical and clinical research and development are reviewed and discussed.

## Highlights

• LAG-3 is a highly important next-generation immune checkpoint molecule.
• Ninety-seven clinical trials are evaluating at least 16 LAG-3-targeting molecules.
• Here we identify preclinical and clinical studies conducted involving LAG-3.
• Bispecific LAG-3 molecules are being developed, showing strong capacities.
• LAG-3/PD-1 co-blockade is demonstrating encouraging results.

## Introduction

Lymphocyte activation gene 3 (LAG-3, CD223) is a cell surface inhibitory receptor that regulates a wide range of T-cell effector functions.^1^, ^2^, ^3^, ^4^, ^5^ LAG-3 plays similar roles to other immune checkpoint molecules such as programmed cell death protein 1 (PD-1) and cytotoxic T-cell lymphocyte antigen-4 (CTLA-4). LAG-3 is expressed by T cells, some activated B cells, plasmacytoid dendritic cells (DCs) and neurons and subjected to epigenetic regulation.^6^^,^^7^ In addition, LAG-3 in activated T cells delivers co-stimulatory signals to DCs, licensing them to produce interleukin-12p70 (IL-12p70).^8^^,^^9^

LAG-3 ligands include major histocompatibility complex (MHC)-II, galectin-3 (Gal-3) and fibrinogen-like protein 1 (FGL1).^10^, ^11^, ^12^, ^13^ MHC-II is considered the canonical ligand.^3^^,^^4^ LAG-3 binds to MHC with higher affinity than CD4, disrupting CD4–MHC-II interactions.^11^^,^^14^ LAG-3 binding induces MHC-II signal transduction in DCs, activating phospholipase C γ2, p72syk, PI3K/AKT, p42/44 and p38 protein kinase.^15^ Engagement with Gal-3 and FGL1 also exerts T-cell inhibitory functions possibly by other means. Gal-3 can be highly expressed on tumor cells and activated T cells; it is required for CD8 T-cell and plasmacytoid DC suppression.^12^^,^^16^, ^17^, ^18^ LAG-3 binding to FGL1 is non-redundant to MHC-II binding, and contribute to poor responses to anti-PD-1/anti-programmed death-ligand 1 (PD-L1) immunotherapies.^13^ In addition, the Dendritic Cell-Specific Intercellular adhesion molecule-3-Grabbing Non-integrin family member LSECtin acts as an LAG-3 ligand in melanoma cells, inhibiting antitumor T-cell responses by reducing the expression of CDK2, CDK4 and CDK6.^19^ LAG-3 inhibits T-cell receptor (TCR) signal transduction by association to the TCR–CD3 complex.^20^ However, the exact mechanisms of intracellular negative signal transduction are still uncharacterized.^5^^,^^21^, ^22^, ^23^

Elevated LAG-3 expression is considered a T-cell exhaustion marker, associated to immune homeostasis disruption in a broad spectrum of human diseases.^6^ Importantly, LAG-3 and PD-1 co-expression in T cells is a biomarker of strong T-cell dysfunctionality in cancer and it is associated with resistance to anti-PD-1/anti-PD-L1 immunotherapies.^24^, ^25^, ^26^, ^27^, ^28^, ^29^, ^30^ PD-1 and LAG-3 co-blockade increases many T-cell antitumor activities.^26^^,^^28^^,^^31^, ^32^, ^33^ Several immunotherapies targeting LAG-3 are at various stages of clinical development.^6^ Here, we review the publically available data on LAG-3-targeted therapy.

## Preclinical and clinical development of lag-3-targeted therapy

**Figure 1:**
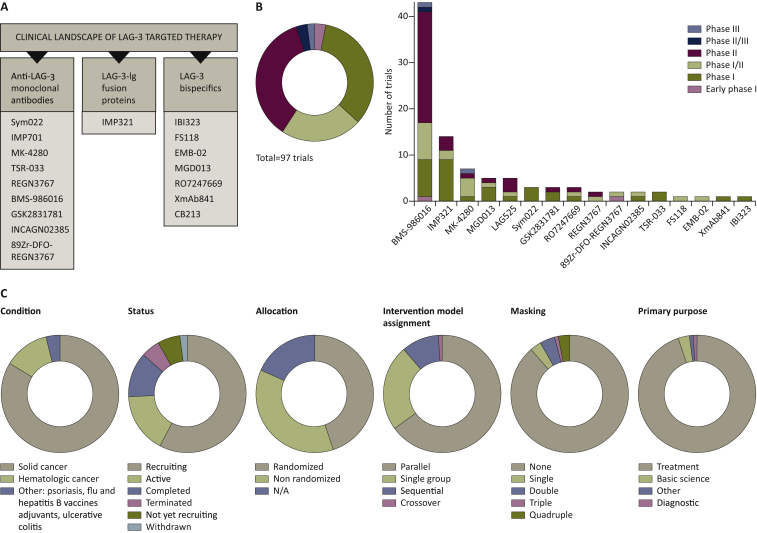
**Detailed analysis of the lymphocyte-activated gene 3 (LAG-3)-targeted therapy clinical landscape.** (A) Current LAG-3-targeted therapies can be categorized into three subtypes: anti-LAG-3 monoclonal antibodies, LAG-3–immunoglobulin (Ig) fusion proteins and LAG-3 bispecifics, as indicated in the figure. Some examples of the tested therapeutic drugs are listed. (B) Categorization of LAG-3 clinical trial phases. On the left, a pie chart with the distribution of LAG-3-targeting trials. On the right, a bar chart indicating the clinical phase distributions for the indicated LAG-3-targeted drugs. (C) Pie charts with categorization of LAG-3 clinical trials by the studied pathologies, status and study design as indicated (allocation, intervention model assignment, masking and primary purpose).

Sixteen LAG-3-targeted therapies are tested at 97 clinical trials by Bristol-Myers Squibb (BMS-986016), Regeneron Pharmaceuticals (REGN3767 and 89Zr-DFO-REGN3767), Merck (MK-4280), Novartis (LAG525), Tesaro (GSK) (TSR-033), Symphogen (Sym022), GlaxoSmith (GSK2831781), Incyte Biosciences International Sàrl (INCAGN02385), Prima BioMed/Immutep (IMP321), MacroGenics (MGD013), F-Star (FS118), Hoffmann-La Roche (RO7247669), Shanghai EpimAb Biotherapeutics (EMB-02), Xencor (XmAb841) and Innovent Biologics (IBI323). Figure 1 summarize the publically available data (Supplementary Table 1, available at https://doi.org/10.1016/j.iotech.2022.100079). These therapies are categorized into monoclonal antibodies, soluble LAG-3–immunoglobulin (Ig) fusion proteins and anti-LAG-3 bispecific drugs (Figure 1A). With the exception of the IgG1 antibody etigilimab, most anti-LAG-3 monoclonal antibodies are fully humanized IgG4 monoclonal blocking antibodies. IMP321 is the only soluble recombinant LAG-3 clinically studied. Additionally, bispecific anti-LAG-3-targeted drugs are being studied with very promising results, especially dual PD-1/LAG-3 blockade. Preliminary evidence from clinical trials is providing encouraging results for the treatment of cancers in terms of efficacy, safety, tolerance and pharmacokinetics.

Most trials are phase I (34), I/II (21) and II (35). Few of them are early phase I (2) and phase II/III (3) trials, and only two of them have reached phase III for BMS-986016 (NCT05002569) and MK-4280 drugs (NCT05064059) (Figure 1B). All of them are interventional and investigational trials, still active and recruiting, and considered as applicable clinical trials by the Food and Drug Administration Amendments Act of 2007 (Figure 1C). Most are open-label, randomized and parallel studies (Figure 1C). The treated solid tumors are varied and summarized in Supplementary Table 1. Treated hematologic malignancies include lymphoma, myeloma and leukemia among others. Interestingly, some LAG-3 inhibitors are also being tested for psoriasis (NCT02195349), ulcerative colitis (NCT03893565), hepatitis B (NCT00354861) and influenza vaccines (Phase I Study of IMP321 Given Alone; https://clinicaltrials.gov/ct2/show/NCT00354263). As LAG-3 is involved in the pathogenesis of a wide range of non-neoplastic disease, it could be a therapeutic target for the treatment of selected pathologies. Of note, a recent controversial study with therapeutic anti-LAG-3 antibodies showed that LAG-3 is not expressed in human and murine neurons and does not modulate α-synucleinopathies.^34^ LAG-3 expression is included in several clinical trials as a biomarker of disease status (Supplementary Table 2, available at https://doi.org/10.1016/j.iotech.2022.100079).

### Anti-LAG-3 monoclonal antibodies

#### BMS-986016, relatlimab

BMS-986016 fully human monoclonal antibody is an anti-LAG-3 fully human monoclonal IgG4-κ antibody, which was the first LAG-3 blocker to be clinically developed.^35^ Relatlimab binds human LAG-3 with high affinity (Kd = 0.12-0.5 nM) and inhibits its binding to MHC-II (WO 2015116539 A1)*.*^36^^,^^37^ Relatlimab is currently being evaluated in phase I, II, II/III and III trials in solid and hematological malignancies, alone or in combination with anti-PD-1/PD-L1 drugs^6^^,^^38^ (Supplementary Table 1). Preliminary data are supporting its use either alone or in combination. It is relatively well tolerated and shows clinical efficacy.^14^^,^^39^ Genomic and immunological differences were found between responder patients and non-responders in a combo treatment with anti-PD-1.^40^ The authors monitored interferon (IFN)-γ cytokine concentrations in real time in tumor tissue biopsies, while evaluating the safety of anti-LAG-3/anti-PD-1 combination.^41^ Relatlimab and nivolumab (anti-PD-1) combination helps in overcoming treatment resistance. For instance, preliminary results (NCT01968109; NCT03470922) showed promising initial efficacy, safety profile, well tolerability and antitumor activity in patients with melanoma in patients progressing to anti-PD-1/PD-L1 monotherapy.^36^^,^^42^^,^^43^ Relatlimab also restores anti-leukemic T- and natural killer (NK) cell-mediated responses in patients with chronic lymphocytic leukemia. Relatlimab induces leukemic cell depletion, enhances NK and antibody-dependent cytotoxicities and promotes T-cell tumor necrosis factor (TNF)-α, IFN-γ and IL-2 cytokine.^44^

Recent phase III data on relatlimab and nivolumab combo versus nivolumab in untreated advanced melanoma showed greater benefit in progression-free survival: 47.7% at 12 months [95% confidence interval (CI), 41.8% to 53.2%] with the PD-1/LAG-3 combination as compared with 36.0% (95% CI, 30.5% to 41.6%) with the anti-PD-1 monotherapy.^45^^,^^46^ The combo showed good toxicity profiles.

#### REGN3767, fianlimab

REGN3767 is an IgG4 fully human, hinge-stabilized, high-affinity, monoclonal antibody developed by Regeneron Pharmaceuticals. It was obtained from VelocImmune mice containing human Ig gene segments.^47^^,^^48^ Fianlimab blocks LAG-3 binding to MHC class II, activating T cells and enhancing cytotoxic T-cell-mediated tumor cell lysis (https://www.cancer.gov/publications/dictionaries/cancer-drug/def/fianlimab). REGN3767/cemiplimab (anti-PD-1) combo demonstrated good antitumor activities both *in vitro* and in the human PD-1xLAG-3-knockin mice model.^49^ Increased activation of tumor-specific T cells was observed, promoting T-cell-mediated immunity. In addition, REGN3767 showed favorable pharmacokinetics and toxicology in cynomolgus monkeys.^49^ Two clinical trials are investigating REGN3767 alone and in combination with anti-PD-1 inhibitors (NCT03005782, NCT01042379). In a phase I, open-label, dose-escalation and cohort expansion first-in-human clinical trial, the combination showed a safety profile similar to other immune checkpoint inhibitors (ICIs) (NCT03005782). Activity and pharmacodynamics were also examined. Preliminary data suggested a dose-dependent expansion of PD-1-expressing memory T-cell subsets by REGN3767/cemiplimab combination. Early efficacy was detected, suggesting that REGN3767 exerts antitumor activity across several tumor types. Thus, a fixed dose was selected for further evaluation.^50^ Fianlimab and cemiplimab combo showed a similar safety profile to cemiplimab alone, with one exception, and a clinical activity similar to anti-PD-1/anti-CTLA-4 combination in melanoma patients but with reduced treatment-emergent adverse events (TEAEs).^51^ Objective response rate (ORR) was 63.6% (3 complete responses and 18 partial responses) for anti-PD-L1-naïve patients and 13.3% (1 complete response and 1 partial responses) for anti-PD-L1-experienced patients. The REGN3767/cemiplimab combo is being evaluated in a phase II adaptively randomized clinical trial for breast cancer^52^ (NCT01042379).

#### 89Zr-DFO-REGN3767, fianlimab tracer

Anti-LAG-3 antibodies are being used for positron emission tomography (PET) scanning as a diagnostic method.^53^ 89Zr-DFO-REGN3767 is an anti-LAG-3 PET imaging tracer that integrates the anti-LAG-3 REGN3767 antibody labeled with zirconium,^54^ used for monitoring therapy response to anti-LAG-3 treatment. This trial has several aims apart from establishing safety, pharmacokinetics, dosing and timing for PET scanning as a diagnostic method. The objectives include tumor targeting, determination of 89Zr-DFO-REGN3767 biodistribution and dosimetry, optimal time for imaging and tumor uptake after drug administration, evaluation of tumor uptake of the 89Zr-DFO-REGN3767 and correlation with LAG-3 expression (NCT04566978). This study is being carried out in early phase I and phase II imaging clinical trials for solid and hematologic cancer (NCT04706715, NCT04566978).

#### Sym022

Sym022 is a recombinant, Fc-inert, fully human, monoclonal antibody developed by Symphogen that blocks LAG-3/MHC-II binding. This antibody binds with high affinity to human and cynomolgus monkey LAG-3 and increases T-cell cytokine production.^55^ Three phase I dose-escalation and dose-expansion clinical trials are testing Sym022 for cancer treatment, alone or in combination with Sym021 (anti-PD-1) and Sym023 (anti-T-cell immunoglobulin and mucin domain-3) (NCT03489369, NCT04641871, NCT03311412). Studies in preclinical models have shown that Sym021, Sym022 and Sym023 combinations provide synergistic antitumor activities.^56^^,^^57^

#### GSK2831781, IMP731

GSK2831781 is a humanized anti-LAG-3 monoclonal IgG1 antibody developed by GlaxoSmithKline (GSK), and derived from Immutep’s IMP731 antibody. This antibody depletes LAG-3-expressing activated T cells in immuno-inflammatory disorders. Two phase I clinical trials are evaluating safety, tolerability, pharmacokinetics and pharmacodynamics for the treatment of psoriasis (NCT03965533, NCT02195349). A phase II clinical trial has been terminated in ulcerative colitis (NCT03893565). These trials were interrupted based on the assessment of clinical data as part of an interim analysis conducted in consultation with the Data Review Committee of the trial^58^ (NCT03893565). Further reporting is being conducted on the efficacy and safety data, although GSK and Immunotep’s collaboration remains in place (https://pipelinereview.com/index.php/2021012277234/Antibodies/Ulcerative-Colitis-Phase-II-Study-of-GSK2831781-Discontinued.html). Preliminary results showed that GSK2831781 is pharmacologically active with a tolerable safety profile, and provides early evidence of improvement in psoriasis.^59^ GSK2831781 treatment reduced pro-inflammatory gene expression (*IL-17A*, *IL-17F*, *IFNγ*, and *S100A12*), and up-regulated genes associated with skin barrier functions (*CDHR1*).

#### INCAGN02385

INCAGN02385 is an Fc-engineered IgG1-κ monoclonal antibody developed by Incyte Corporation, which blocks LAG-3 binding to MHC-II, enhancing T-cell responsiveness to TCR stimulation. Studies in cynomolgus monkeys showed acceptable tolerability and pharmacokinetics, with cross-reactivity to cynomolgus monkey LAG-3.^60^ INCAGN02385 has been evaluated alone in a completed phase I clinical trial (NCT03538028), and it is being currently studied in a phase I/II clinical trial in combination with INCMGA00012 (anti-PD-1) and INCAGN02390 (anti-TIM-3) in patients with advanced malignancies (NCT04370704).

#### TSR-033

TSR-033 is an anti-LAG-3 high-affinity human IgG4 monoclonal antibody developed by Tesaro (GSK) (PMID: 30587557), and binds with high affinity to human LAG-3.^61^ Complementary determining regions (CDRs) from the original murine antibody were grafted within the germline frameworks of the human ortholog, followed by *in vitro* somatic hypermutation with mammalian cell surface display for further selection of high-affinity variants.^62^^,^^63^ TSR-033 demonstrated antitumor activities in preclinical models.^61^ TSR-033 in combination with anti-PD-1 increased IL-2 production by activated CD4 T cells and improved efficacy. The combo treatment increased total and intra-tumor T-cell proliferation and stimulation, and reduced tumor-associated macrophages. Two dose-escalation phase I clinical trials (NCT03250832, NCT02817633) are investigating TSR-033 alone and in combination with anti-PD-1 antibody. Preliminary data showed good tolerability and safety profiles (NCT03250832).

#### LAG525, IMP701, ieramilimab

LAG525 is a humanized IgG4 monoclonal antibody developed by Novartis which blocks LAG-3 binding to MHC-II [concentration that causes 50% inhibition of growth (IC_50_) 5.5 nM].^64^ Five trials are studying LAG525 in several phases, administered alone or in combination with anti-PD-1 inhibitors. Preliminary data show that LAG525 exhibits good safety profile and pharmacokinetics, as well as promising antitumor activity.^65^^,^^66^ The combination was also well tolerated.^67^ A phase I/II study in combination with spartalizumab (PDR001) reported well-tolerated dose-escalation results and good antitumor activities.^65^ Later, a phase II was conducted in patients with solid or hematologic malignancies that relapsed or were refractory to non-immunotherapies.^66^ Promising antitumor activity for neuro endocrine tumors, small-cell lung cancer and diffuse large B-cell lymphoma (DLBCL) were also found. LAG525 combination with spartalizumab (anti-PD-1) exhibited good safety profiles and antitumor activities in melanoma, renal cell cancer and mesothelioma previously treated with PD-1/PD-L1 blockers, suggesting that LAG-3 blockade counteracts prior resistance to these treatments^67^ (‘NCT02460224’).

#### MK-4280, favezelimab

MK-7684 is a humanized IgG4 monoclonal anti-LAG-3 monoclonal antibody developed by MERCK. Favezelimab increased cytokine (IFN-γ, IL-2, IL-8 and TNF-α) and chemokine (CCL4, CXCL10 and CCL22) production in T cells, and CD69, CD44, CD25, XCL1, GZMB and Nuclear factor of activated T-cells up-regulation.^68^ Seven clinical trials are evaluating MK-4280 at various clinical stages. Preliminary findings demonstrated good safety and efficacy profiles, and manageable tolerability when administered alone or in combination with other immune checkpoint blockade agents (NCT05064059).^69^ A phase I/II first-in-human trial is evaluating its safety and efficacy in combination with pembrolizumab (anti-PD-1).^70^ Preliminary results showed that favezelimab alone or in combination presents good safety profile.^71^ The combo showed better antitumor activity than monotherapy with an ORR of 6.3% (one complete response and four partial responses). Treatment-related adverse events were 65% with favezelimab and 65.2% in combination with pembrolizumab, most commonly fatigue, fever and nausea.

### Soluble LAG-3–Ig fusion proteins

#### IMP321, eftilagimod alpha (efti)

IMP321, a soluble LAG-3 molecule, is a natural, high-affinity, human and murine MHC-II agonist (hLAG-3Ig) developed by Immutep. It is a dimeric recombinant molecule with LAG-3 extracellular domains fused to a human immunoglobulin Fc region. IMP321 is an atypical ICI as it targets antigen-presenting cells (APCs) such as DCs. Indeed, natural soluble LAG-3 is associated to protective resistance against tuberculosis and favorable outcome, and to improved disease-free and overall survival rates in some breast cancers expressing estrogen or progesterone receptors.^72^^,^^73^ IMP321 was firstly developed as an immune modulator for vaccines. It is being evaluated as a treatment for cancer as well. IMP321 increases T-cell responses and vaccine immunogenicity to various diseases, specifically generating type 1 tumor-specific immunity by enhancing the release of Th1 cytokines by APCs.^74^ IMP321 demonstrated good safety and tolerability, and increased Th1 responses to influenza vaccine.^75^ IMP321 enhanced T-cell responses against an alum-non-absorbed recombinant hepatitis B surface antigen, inducing humoral and T-cell-mediated immunity.^76^^,^^77^ IMP321 recruits and activates effector innate and adaptive immune cells.^78^ Indeed, IMP321 enhanced T-cell proliferation and induced a full Tc1-activated phenotype characterized by IFN-γ, TNF-α, IL-1β, IL-6, CCL4, CCL5 and CCL2 production. Additionally, MP321 treatment programed myeloid cells to produce CCL4 and TNF-α, and CD8 and NK cells to produce IFN-γ and TNF-α.^78^ These effects induce monocyte-derived DC maturation and migration *in vitro,* and stimulate naïve and Th1 responses.^77^^,^^79^^,^^80^ IMP321 delayed tumor growth and enhanced tumor rejection, through tumor-specific CD8 effector and memory responses.^81^^,^^82^ Indeed, IMP321 possesses strong adjuvant activities, expanding tumor and virus antigen-specific T cells and activating APCs, generating long-lasting immunity.^74^ Fourteen clinical trials are testing IMP321 at phase I, I/II and II stages in infectious and malignant diseases. It is demonstrating good efficacy, activity and safety, with appropriate pharmacodynamics in combination with other antitumor therapies.^83^, ^84^, ^85^, ^86^, ^87^ It has been used as an adjuvant in combination with chemotherapy and autologous adoptive T-cell transfer, achieving long-lasting antitumor immunity. Stronger expansion of antigen-specific effector CD8 T cells and reduced expansion of regulatory T cells were observed.^88^ A phase I trial of Melanoma antigen recognized by T cells 1 peptide vaccination plus IMP321 in patients receiving autologous peripheral blood mononuclear cell (PBMC) transfer after lymphodepleting chemotherapy showed that vaccination induced a durable antitumor immune cell response.^88^ CD8 T cells did not show exhaustion phenotypes. IMP321 also showed good tolerability and toxicity profiles as front-line therapy in combination with gemcitabine in patients with advanced pancreatic adenocarcinoma.^89^ No significant differences were observed in monocytes (CD11b+CD14+), DCs (CD11c+), CD4 T and CD8 T cells or in MHC-II, CD80, and CD86 expression between pre- and post-treatment. Vaccination with IMP321 with immunogenic peptides demonstrated good induction of T-cell responses in metastatic melanoma patients.^90^

A phase IIb study with paclitaxel in metastatic breast cancer is being evaluated.^85^^,^^91^ Recent results from the stratum D of the INSIGHT platform trial showed that IMP321 combination with avelumab (anti-PD-L1) is feasible, safe and well tolerated in advanced-stage solid tumors. Of the eight patients enrolled by 2020, 50% progressed, 12.5% had partial responses, 12.5% had stable disease and 25% did not undergo tumor assessment by that time.^87^^,^^92^

### Anti-LAG-3 bispecifics

#### MGD013, tebotelimab

MGD013 is a humanized, hinge-stabilized, IgG4-κ tetravalent bispecific Fc-bearing dual-affinity re-targeting antibody-like (DART®), binding PD-1 and LAG-3 with high affinity. Tebotelimab targets PD-1 and LAG-3-expressing cells and chronically activated T cells. MGD013 is developed by Macrogenetics, and has demonstrated favorable biophysical and manufacturability properties with a prolonged half-life. PD-1/LAG-3 co-blockade caused increased cytokine secretion and enhanced T-cell responses compared to PD-1 or LAG-3 single blockade.^93^ Seven clinical trials are evaluating MGD013 monotherapy and combinations.^94^, ^95^, ^96^, ^97^ Preliminary data show encouraging responses and acceptable pharmacokinetics.^96^ MGD013 dose escalation was well-tolerated with manageable immune-related adverse events, similar to anti-PD-L1. MGD013 monotherapy showed antitumor activity in multiple tumor types, and one complete response after single MGD013 administration in chimeric antigen receptor (CAR)-T-cell therapy. High baseline LAG-3/PD-1 expression and IFN-γ high gene signature (CXCL9, CXCL10, CXC11, STAT1) were associated with objective clinical responses. Furthermore, margetuximab [anti-human epidermal growth factor receptor 2 (HER2)] combination with PD-1xLAG-3 DART® enhanced lytic activity of immune cells. Preliminary results in relapsed or refractory HER2+ solid tumors included an ORR of 42.9%. Further evaluation of MGD013 alone and in combinations is ongoing.^96^^,^^98^ A phase I study tested MGD013 in patients with relapsed or refractory DLBCL, demonstrating good pharmacodynamics, safety profiles and antitumor activities with and without prior CAR-T-cell treatment.^93^

#### FS118

FS118 is a first-in-class human tetravalent, full-length human IgG1, anti-LAG-3/PD-L1 bispecific antibody developed by F-star Therapeutics. This bispecific blocks both PD-L1 and LAG-3 with high affinity, and demonstrates comparable activity to single blockades.^99^ FS118 overcomes PD-L1- and LAG-3-mediated inhibition of T-cell activation and effector functions *in vitro*. In addition, its surrogate mouse version inhibits tumor growth *in vivo* and contributes to LAG-3 and PD-L1 shedding by the activities of A Disintegrin And Metalloproteinase 10 and A Disintegrin And Metalloproteinase 17.^99^^,^^100^ Its binding can overcome PD-L1-mediated compensatory up-regulation of LAG-3. FS118 monotherapy is being evaluated in advanced malignancies in a phase I/II clinical trial (NCT03440437). Preliminary data suggest good tolerability and pharmacodynamics, and early signs of clinical efficacy.^101^^,^^102^ FS118 potently activates primary human T cells *in vitro* and inhibits tumor growth in carcinoma models (https://www.nature.com/articles/d43747-020-00181-6). A phase I study in patients with prior resistance to immune checkpoint therapy exhibited good pharmacodynamic and pharmacokinetic profiles. The treatment was well tolerated, with signs of improved clinical outcomes, consistent with preclinical data.^101^^,^^102^ Furthermore, FS119 showed early signs of clinical efficacy with long-term disease control in patients with previous acquired resistances.

#### RO7247669

RO7247669 is an anti-PD-1-LAG-3 bispecific antibody developed by Hoffmann-La Roche that binds to PD-1 and LAG-3, blocking their inhibitory pathways. RO7247669 is being evaluated in three clinical trials at the recruiting phase for the treatment of solid tumors (NCT04524871, NCT04785820, NCT04140500).

#### EMB-02

EMB-02 is an anti-PD-1-LAG-3 bispecific antibody developed by EpimAb Biotherapeutics, which blocks their binding to PD-L1, MHC-II and FGL-1. Its binding also induces PD-1 and LAG-3 degradation, and preclinical data show improvement for the treatment of tumor models resistant to PD-1 blockade monotherapies. One phase I/II clinical trial is currently recruiting patients to evaluate EMB-02 in advanced solid tumors (NCT04618393).

#### XmAb841, pavunalimab

XmAb841 is an anti-CTLA4-LAG-3 bispecific antibody developed by Xencor, which enhances T-cell stimulation and proliferation. Its structure consists of bispecific Fc domains as a scaffold between the two binding domains, conferring stability, ease of purification and manufacture. The Fc domains lack Fcγ receptor binding. This structure promotes heterodimer formation and a long-circulating half-life. XmAb841 enhanced human T-cell activation. This bispecific also enhanced allogeneic antitumor activity and can be combined with anti-PD-1 blockade to promote triple checkpoint blockade (https://investors.xencor.com/static-files/3761fc99-37a0-486e-a5b7-ad94f17eb446; https://investors.xencor.com/static-files/5042016b-70bd-40f5-abf7-862ddd759986; https://investors.xencor.com/static-files/f388d30a-3d0d-4a69-9a43-876a3b38f79f). This biospecific molecule enhanced IL-2 production *in vitro* (https://investors.xencor.com/static-files/f388d30a-3d0d-4a69-9a43-876a3b38f79f). There is only one phase I clinical trial at recruitment stage in selected advanced solid tumors (NCT03849469).

#### IBI323

IBI323 is an LAG-3/PD-L1 bispecific antibody developed by Innovent Biologic, which preserves the properties of the parental antibodies. IBI323 blocks engagement of PD-1 with PD-L1 and CD80, and of LAG-3 with MHC-II.^103^ This bispecific activates T cells, enhancing their activation by crosslinking PD-L1+ APCs with LAG-3+ T cells, and promoting antitumor activities in humanized mouse models. Treatment with IBI323 increased numbers of intra- and extra-tumor T cells, and tumor antigen-specific T cells.^103^ A phase I clinical trial is currently studying IBI323 in advanced malignancies.

#### CB213

Crescendo Biologics Ltd recently announced a clinical development partnership with Cancer Research UK to progress the bispecific PD-1xLAG-3 antagonist Humabody® CB213 into a phase I clinical trial targeting solid tumors (https://www.businesswire.com/news/home/20200505005080/en/Crescendo-Biologics-and-Cancer-Research-UK-sign-Clinical-Development-Partnership-to-develop-CB213-a-novel-bispecific-Humabody%C2%AE-therapeutic). Its structure consists of human nanobodies (VH human bodies) with an asymmetric 2 : 1 binding format using bivalent LAG-3-binding human bodies coupled to a monovalent PD-1 human body. CB213 is a half-life extended version delivering simultaneous PD-1 and LAG-3 checkpoint blockade specifically targeted toward highly dysfunctional LAG-3+ PD-1+ double-positive T cells.^26^^,^^104^ This approach has been designed to deliver safer, more effective therapeutic interventions in patients with cancers resistant or refractory to PD-1 monotherapies. In preclinical testing, CB213 has demonstrated a potent dual checkpoint blockade activity with the ability to revert the dysfunctionality of patient-derived T cells. Antitumor efficacy was characterized by a potent inhibition of tumor growth and enhancement of tumor antigen-specific CD8+ cytotoxic T cells.^105^

## Conclusions

LAG-3 is one of the most important next-generation immune checkpoint molecule. Currently, 97 clinical trials are evaluating at least 16 LAG-3-targeting molecules (Supplementary Table 1 and Figure 1). Only two trials have reached phase III, one by BMS and the other by Merck, which will be a major part in developing clinical LAG-3 targeting. Phase III studies in melanoma and colorectal cancer are demonstrating encouraging results. Here, we have discussed the positive results of these phase III trials. Other LAG-3-targeting drugs were developed later and have not yet progressed to phase III trials. Most of the trials test LAG-3-antagonistic molecules including combinations with other ICIs. Next-generation bispecifics have been developed, showing stronger capacities for specific dual targeting of LAG-3 with other ICIs. This is significant, because simultaneous co-expression of LAG-3 with other immune checkpoint molecules is a distinguishing feature of highly dysfunctional T cells in cancer patients. LAG-3-targeting cancer immunotherapies have demonstrated good safety profiles, tolerability and adequate pharmacokinetics and pharmacodynamics, with promising antitumor efficacy. In addition, LAG-3 regulates a diversity of biological mechanisms and its use as a cancer target keeps on holding relatively high promises. However, there are never guarantees on clinical efficacies and breakthroughs. Only time will tell whether PD-1 and LAG-3 co-blockade will counteract resistance to current immunotherapies.
